# Exactitud de localizadores apicales integrados en motores endodónticos según el tipo de lima utilizada: comparación entre limas manuales y mecanizadas

**DOI:** 10.21142/2523-2754-1303-2025-254

**Published:** 2025-08-31

**Authors:** María Alejandra Portillo Martínez, Luz Duarte, Milagros Vega, Constanza Real Aparicio

**Affiliations:** 1 Facultad de Odontología, Universidad del Pacífico. Asunción, Paraguay. portilloale5@gmail.com, luzbelladuar@gmail.com, Milagrosvega753@gmail.com, constanzareal05@gmail.com Asunción Paraguay portilloale5@gmail.com luzbelladuar@gmail.com Milagrosvega753@gmail.com constanzareal05@gmail.com

**Keywords:** localizador apical, longitud radicular, motores endodónticos, apical locator, root length, endodontic motors

## Abstract

**Objetivos:**

El presente estudio piloto de tipo observacional analítico in vitro evaluó la exactitud de tres localizadores apicales (Root ZX mini/Morita, Endo Radar Plus/Woodpecker y Endo Motor SCM-011/Fanta) para la determinación de la longitud radicular, comparando mediciones realizadas con limas manuales y mecanizadas con respecto a un valor de referencia obtenido por tomografía computarizada de haz cónico (CBCT).

**Materiales y métodos:**

Se utilizaron 60 dientes humanos permanentes, distribuidos aleatoriamente en tres grupos según el localizador apical utilizado. Se midió la longitud del conducto con limas manuales (sin motor) y con limas mecanizadas accionadas por motor con función de localizador integrada. Se calcularon los errores absolutos en relación con el valor de referencia y se aplicaron pruebas t de Student y Anova (p < 0,05).

**Resultados:**

Los resultados mostraron que las limas manuales presentaron un menor error absoluto promedio (0,253 mm) que las mecanizadas (0,423 mm), con diferencias significativas (p < 0,0001). Entre los localizadores, utilizando limas manuales, Endo Radar mostró mayores errores absolutos comparado con Root ZX mini (p = 0,021) y Endo Motor SCM-011 (p = 0,004), sin diferencias significativas entre Root ZX mini y Endo Motor SCM-011 (p = 1,000). Con limas mecanizadas, los errores fueron similares entre los tres localizadores (p = 0,768).

**Conclusión:**

Las limas manuales proporcionaron mediciones más exactas que las mecanizadas. Los localizadores Root ZX mini y Endo Motor SCM-011 mostraron mayor exactitud con limas manuales. Con las limas mecanizadas, no se observaron diferencias significativas entre los localizadores. Estos resultados preliminares revelan la influencia del tipo de instrumento y la modalidad de activación del localizador en la exactitud de la medición radicular.

## Abstract (en)

**Objectives:**

This pilot in vitro analytical observational study evaluated the accuracy of three apex locators-Root ZX mini (Morita), Endo Radar Plus (Woodpecker), and Endo Motor SCM-011 (Fanta)-in determining root canal length, comparing measurements obtained using manual and mechanized files against a reference value obtained through cone-beam computed tomography (CBCT).

**Materials and Methods:**

Sixty permanent human teeth were randomly distributed into three groups according to the apex locator used. Root canal length was measured with manual files (without motor) and with mechanized files driven by endodontic motors with integrated apex locator functionality. Absolute errors were calculated in relation to the reference value, and statistical analyses were performed using Student's t-test and ANOVA (p<0.05).

**Results:**

Manual files showed a lower mean absolute error (0.253 mm) than mechanized files (0.423 mm), with statistically significant differences (p < 0.0001). Among the apex locators, when using manual files, Endo Radar exhibited higher absolute errors compared to Root ZX mini (p = 0.021) and Endo Motor SCM-011 (p = 0.004), with no significant differences between Root ZX mini and Endo Motor SCM-011 (p = 1.000). With mechanized files, the error rates were similar across the three devices (p = 0.768).

**Conclusion:**

Manual files provided more accurate root length measurements than mechanized ones. The Root ZX mini and Endo Motor SCM-011 showed greater accuracy when used with manual files. These preliminary findings highlight the influence of file type and activation mode of the apex locator on the accuracy of root length determination.

## INTRODUCCIÓN

Para un tratamiento exitoso en la endodoncia actual, la utilización de localizadores apicales se ha convertido en una herramienta indispensable, ya que estos dispositivos permiten determinar con exactitud la longitud del conducto radicular, lo que es crucial para realizar una obturación adecuada y evitar complicaciones postoperatorias, lo que contribuye a la reducción de los fracasos durante el tratamiento ^1^. La longitud de trabajo (LT) corresponde a la distancia desde la referencia al punto final apical de los procedimientos endodónticos que, idealmente, debe corresponder a la constricción apical (CA) ^2^. Antiguamente, se utilizaban mediciones radiográficas para la determinación de la longitud del diente; no obstante, estas pueden proporcionar mediciones incorrectas debido a las variaciones en la posición del foramen. Varios estudios han demostrado que los localizadores apicales son más confiables que las mediciones radiográficas ^3^.

En la actualidad, se considera como estándar de referencia en exactitud al Root ZX mini, un localizador apical de tercera generación ^4^, y se basa en el “método de ratio”, que utiliza frecuencia dual e impedancia proporcional. Para ello, emplea las raíces cuadradas medias de las impedancias medidas a frecuencias de 0,4 y 8,0 kHz. Estas impedancias se miden por separado y simultáneamente, y se comparan con los valores de referencia almacenados en la memoria del dispositivo ^5^. Este dispositivo, además de estar diseñado para ser portátil, se destaca que es menos sensible al contenido intracanal. El Root ZX mini cuenta con calibración automatizada, resistencia a golpes y tres configuraciones de memoria programadas ^6^. Sin embargo, existe poca información en la literatura sobre la exactitud del Root ZX mini para medir la longitud del diente funcionando a la par con el motor endodóntico.

La práctica endodóntica se ha simplificado gracias a una innovación tecnológica que son los localizadores apicales integrados al motor de endodoncia. Estos equipos combinan la funcionalidad de un localizador apical con la exactitud y el control de un motor de endodoncia en una sola unidad, lo que permite al endodoncista realizar procedimientos más eficientes y precisos ^7^. Gracias a la integración de un localizador apical en el motor de endodoncia, el profesional puede determinar, simultáneamente y con exactitud, la longitud del conducto radicular y controlar tanto la velocidad como la dirección de la lima. Hay estudios que aseguran que esta combinación de tecnologías reduce la posibilidad de errores en la medición de la longitud de trabajo, mejora la eficiencia del tratamiento y reduce el tiempo de trabajo ^8^.

Debido a la amplia variedad de localizadores apicales y motores endodónticos disponibles en el mercado, surge la interrogante de si es posible combinar estos dispositivos en un único equipo que funcione como motor endodóntico con localización apical, manteniendo un nivel de exactitud comparable al de los dispositivos individuales ^7^.

Uno de los dispositivos que encontramos en el mercado es el Endo Motor SCM-011 (Fanta Dental, Shanghái, China), que es un localizador apical basado en 2 frecuencias con un módulo opcional para instrumentación rotatoria, lo cual le permite funcionar como una pieza de mano de baja velocidad, como localizador apical o una combinación de ambos. El dispositivo, además, cuenta con una función de inversión apical automática (AAR) que detiene e invierte la rotación de la lima cuando la punta alcanza el límite apical especificado.

Otro motor que encontramos en el mercado y que trae integrado un localizador apical es el Endo Radar Plus Motor (Woodpecker Dental, China), un localizador apical de tercera generación que trabajan con el método ratio. Estos dispositivos utilizan múltiples frecuencias para determinar la longitud del conducto radicular. Podemos ver como una ventaja para el profesional el hecho de que elimina la necesidad de dispositivos individuales para determinar la longitud de trabajo y la conformación de los conductos radiculares, lo cual disminuye el tiempo en el consultorio ^9^.

En la última década, numerosos estudios han evaluado la efectividad y exactitud de los localizadores apicales en comparación con las técnicas convencionales de medición. Sin embargo, la literatura sobre la exactitud de los localizadores apicales integrados a motores endodónticos disponibles en el mercado es limitada. Por este motivo, el objetivo principal de la presente investigación fue de comparar *in vitro* la exactitud de los localizadores apicales integrados en motores endodónticos, utilizando limas manuales y mecanizadas.

## MATERIALES Y MÉTODOS

El presente estudio es de tipo observacional analítico, realizado *in vitro*, en el cual se seleccionaron 60 dientes permanentes humanos con ápices completamente desarrollados. Para cada muestra, se obtuvo previamente el consentimiento informado de los individuos, tanto para la extracción dental como para su uso con fines de investigación.

Inicialmente, se realizaron radiografías diagnósticas utilizando la técnica paralela para evaluar las características de las raíces dentales. Se incluyeron raíces de dientes anteriores, premolares y molares, específicamente las raíces distales de molares inferiores y las raíces palatinas de molares superiores. Los criterios de inclusión fueron los siguientes: desarrollo radicular completo, raíces rectas, ausencia de signos de calcificación o reabsorción radicular, conductos sin bifurcación, y ausencia de tratamientos endodónticos previos.

**Figura 1:**
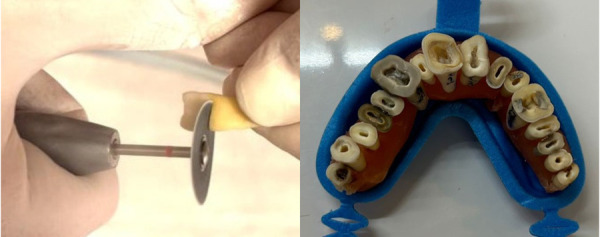
Corte de las piezas a nivel amelocementario.

Las superficies dentales fueron cuidadosamente limpiadas para eliminar residuos de tejido y cálculo mediante un dispositivo ultrasónico (Disco 916D.HP220; Jota, Rüthi, Suiza). Posteriormente, las piezas dentales se cortaron a nivel del límite amelocementario, perpendicular al eje longitudinal del diente, utilizando un disco diamantado (Disco 916D.HP220; Jota, Rüthi, Suiza) y una pieza de mano recta de baja rotación. Este procedimiento permitió exponer el conducto radicular completamente permeable y estableció un punto de referencia nivelado y reproducible (Figura 1). Los dientes se colocaron en un contenedor con alginato (Hydrogum 5, Zhermack, Badia Polesine, Italia), seleccionado como medio electroconductor por su capacidad para permitir mediciones repetitivas y su amplia aceptación en estudios similares ^10^^-^^12^.

Los dientes se codificaron y asignaron aleatoriamente a tres grupos experimentales, según el localizador apical utilizado:

• Grupo 1: Localizador apical Root ZX mini (Morita, Kyoto, Japón) y Motor X Smarth Plus (Dentsply Sirona, Charlotte, Carolina del Norte, EE. UU.), n = 20.
• Grupo 2: Localizador apical Endo Radar Plus Motor (Woodpecker Dental, China), n = 20.
• Grupo 3: Localizador apical Endo Motor SCM-011 (Fanta Dental, Shanghái, China), n = 20.

En el caso del grupo 1, se utilizó el localizador apical Root ZX mini junto a limas manuales. Posteriormente, se empleó el motor X Smart Plus (motor que no incluye función de localizador) únicamente para accionar la lima mecanizada correspondiente, y se mantuvo la medición con el Root ZX mini. En los otros dos grupos (Woodpecker y Fanta), se utilizó la función integrada de motor y localizador con limas mecanizadas.

**Figura 2:**
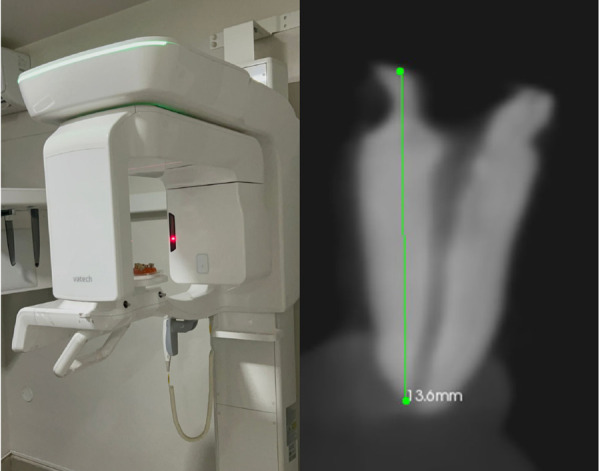
Determinación de la longitud en un corte sagital con el auxilio de un CBCT.

Las piezas dentales se montaron en un molde de cera simulando una arcada dentaria (Figura 1) y se escanearon mediante tomografía computarizada Cone Beam (CBCT) utilizando un Vatech Smart Plus (Modelo PHT-35LHS, Corea), con parámetros estandarizados: 100-240 V, 50-60 Hz, 2.0 KVA, campo de visión de 50 × 50 mm² y tamaño de vóxel de 0,080 mm (Figura 2).

Las imágenes CBCT se analizaron mediante el *software* CS 3D Imaging Software (Carestream Dental LLC, Atlanta, EE. UU.), delimitando y midiendo la distancia entre puntos anatómicos específicos, desde la referencia hasta la salida del foramen. Para garantizar consistencia, los puntos de referencia se marcaron previamente en los dientes con un marcador permanente y se utilizaron para las mediciones con los localizadores apicales.

**Figura 3:**
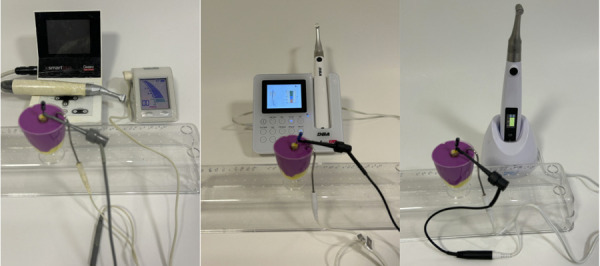
Mediciones de las longitudes radiculares utilizando los localizadores con las limas manuales. A. Root ZX mini (Morita), B. Endo Radar Plus Motor (Woodpecker), C. Endo motor SCM-01 (Fanta Dental).

Las lecturas de la longitud del conducto radicular fueron realizadas por un único operador. Primero se utilizaron las limas manuales, ajustadas al diámetro del conducto de cada diente y con irrigación de hipoclorito de sodio al 2,5%. A continuación, se realizaron las mediciones con limas mecanizadas en función reciprocante, accionadas con el motor endodóntico y conectadas al localizador apical correspondiente (Figuras 3 y 4). 

**Figura 4:**
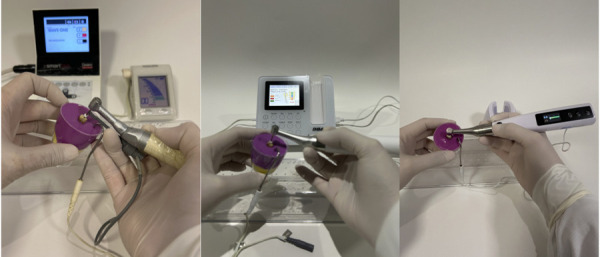
Mediciones de las longitudes radiculares utilizando los localizadores con las limas manuales. A. Root ZX mini (Morita)+ X Smart Plus, B. Endo Radar Plus Motor (Woodpecker), C. Endo motor SCM-01 (Fanta Dental).

Las instrumentaciones se realizaron hasta que el localizador marcó “APEX” o “0”, dependiendo del dispositivo utilizado. Las longitudes obtenidas se midieron con una regla endodóntica milimétrica y se registraron en una hoja de cálculo de Microsoft Excel para su análisis estadístico.

Los datos obtenidos se analizaron mediante métodos estadísticos descriptivos e inferenciales. Inicialmente, se calcularon las medias y desviaciones estándar de las mediciones de longitud radicular realizadas con limas manuales y mecanizadas en cada grupo experimental, así como las diferencias (error absoluto) con respecto al patrón de referencia (medidas obtenidas en las imágenes de CBCT), con el objetivo de evaluar la exactitud de cada grupo de localizadores. Luego de comprobar la normalidad de los datos, fue utilizada la prueba T de Student para muestras pareadas, a fin de comparar los métodos manual y mecanizado de cada tipo de localizador. Seguidamente, para evaluar las diferencias entre los tres tipos de localizadores utilizados, se realizó un análisis de varianza (Anova) de una vía (*post hoc* de Bonferroni). Se estableció un nivel de significancia de p < 0,05. 

El análisis estadístico fue realizado utilizando el *software* Epi Info™.

## RESULTADOS

El presente estudio evaluó *in vitro* la exactitud de tres localizadores apicales, integrados o conectados a motores endodónticos, al determinar la longitud radicular utilizando limas manuales y mecanizadas, en comparación con un patrón de referencia obtenido mediante tomografía computarizada de haz cónico (CBCT). 

### Análisis descriptivo

En el análisis descriptivo general, pudo observarse que las mediciones realizadas con limas manuales mostraron una mayor aproximación a la referencia, con diferencias mínimas y baja variabilidad en los grupos Root ZX mini (Morita) y Endo motor SCM-01 (Fanta). Por otro lado, en el grupo Endo Radar (Woodpecker), aunque la lima manual también presentó una mayor proximidad con la referencia, se observaron discrepancias más evidentes (Tabla 1).

**Tabla 1:** Comparación descriptiva de las mediciones de longitud radicular obtenidas mediante el uso de limas manuales y mecanizadas en los tres localizadores evaluados (mm).

		Cone beam		Lima manual		Lima mecanizada	
		Media	DE	Media	DE	Media	DE
Grupo 1	Root ZX mini (Morita)	15,08	1,95	15,02	1,97	15,00	2,05
Grupo 2	Endo Radar (Woodpecker)	14,41	2,19	14,93	2,28	14,38	2,24
Grupo 3	Endo motor (Fanta)	14,73	1,90	14,79	1,93	14,52	1,82

En cada grupo, el método manual tuvo un menor error promedio que el mecanizado. Las diferencias fueron más pronunciadas en los grupos Woodpecker y Fanta (Tabla 2). 

**Tabla 2:** Errores absolutos calculados para cada grupo.

Grupo	Método	Media	Mediana	DE
Grupo 1	Manual	0,243	0,2	0,255
Morita	Mecanizado	0,327	0,3	0,410
Grupo 2	Manual	0,322	0,3	0,356
Woodpecker	Mecanizado	0,512	0,4	0,621
Grupo 3	Manual	0,174	0,1	0,194
Fanta	Mecanizado	0,428	0,2	0,507

De manera global, el error absoluto promedio es menor para el método manual (0,253) en comparación con el mecanizado (0,423). Además, la dispersión de los errores (desviación estándar) es mayor en el método de utilización de lima mecanizada (Tabla 3).

**Tabla 3:** Error absoluto promedio en la determinación de la longitud radicular según tipo de lima utilizada (manual vs. mecanizada). Se presentan la media, mediana, desviación estándar (DE), valor mínimo y máximo para cada método.

Método	Media	Mediana	DE	Mínimo	Máximo
Manual	0,253	0,2	0,270	0,0	2,7
Mecanizado	0,423	0,3	0,462	0,0	2,8

## COMPARACIÓN ESTADÍSTICA

### Manual vs mecanizado

Se realizó una prueba t pareada para evaluar la diferencia entre los errores absolutos de los métodos manual y mecanizado. Los resultados mostraron un estadístico t de -5,842 y un p-valor menor a 0,0001, lo que permitió rechazar la hipótesis nula. Estos hallazgos evidenciaron que el método manual presenta un error absoluto significativamente menor en comparación con el método mecanizado.

### Entre los tres tipos de localizadores utilizando lima manual

El Anova mostró diferencias estadísticamente significativas entre los tres grupos evaluados (F = 6,574, p = 0,003). Según el análisis *post-hoc* de Bonferroni, se identificó que las diferencias entre el grupo Root ZX mini (Morita) y el grupo Endo Radar (Woodpecker) fueron significativas (diferencia media = -0,34181, p = 0,021). De igual manera, las diferencias entre el grupo Endo Radar (Woodpecker) y el grupo Endo Motor (Fanta) también fueron significativas (diferencia media = 0,40526, p = 0,004). Por otro lado, no se encontraron diferencias significativas entre los grupos Root ZX mini (Morita) y Endo Motor (Fanta) (p = 1,000). Estos resultados indican que el grupo Endo Radar (Woodpecker), utilizando lima manual, presenta errores absolutos mayores en comparación con los otros tipos de localizadores (figura 7).

**Figura 5:**
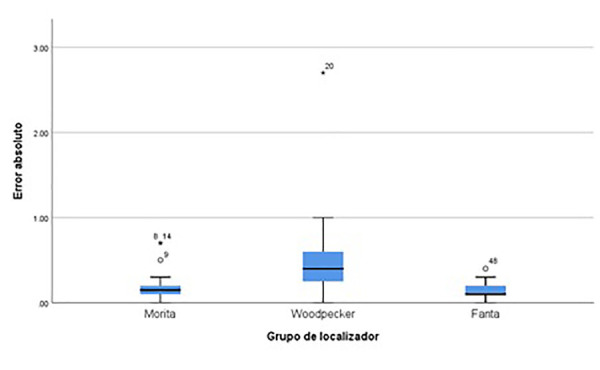
Error absoluto comparado entre los diferentes localizadores utilizando lima manual.

### Entre los tres tipos de localizadores utilizando lima mecanizada

El Anova no reveló diferencias estadísticamente significativas entre los grupos (F = 0,265, p = 0,768). Esto sugiere que el error absoluto utilizando limas mecanizadas es similar en todos los grupos de localizadores estudiados (figura 8).

**Figura 6:**
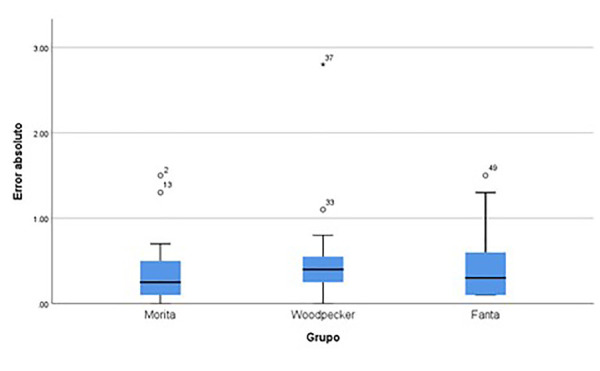
Error absoluto comparado entre los diferentes localizadores utilizando lima mecanizada.

## DISCUSIÓN

Este estudio tuvo como objetivo establecer una comparación *in vitro* de la exactitud entre los localizadores apicales integrados al motor endodóntico usando limas manuales y mecanizadas accionadas con el motor endodóntico para determinar la longitud de trabajo. Varios estudios recientes han evaluado la exactitud de diferentes localizadores apicales *in vitro*^11^^,^^13^ e *in vivo*^14^. 

Para la evaluación de las longitudes dentales, las radiografías periapicales presentan limitaciones en términos de exactitud en comparación con las tomografías computarizadas ^15^. En este estudio se utilizaron mediciones obtenidas mediante CBCT, lo que ofrece varias ventajas significativas, entre las que se incluye su alta exactitud, al proporcionar imágenes tridimensionales de alta resolución que permiten una evaluación exacta de las estructuras dentales, óseas y de los tejidos circundantes. Además, permite evaluar no solo la longitud externa del diente, sino también las estructuras internas y la morfología radicular, información que no se obtiene con otros métodos. Este nivel de detalle es particularmente relevante en contextos donde las mediciones milimétricas son esenciales, como la localización precisa de la salida del foramen. Asimismo, la capacidad de esta técnica para generar datos detallados y confiables optimiza la planificación y el pronóstico de los tratamientos, lo que contribuye a la minimización de errores clínicos ^16^^,^^17^. Con los resultados de este estudio podemos corroborar que las mediciones con los localizadores apicales son comparables a las medidas obtenidas en la CBCT, esto concuerda con varios estudios encontrados en la literatura ^16^^,^^18^^-^^20^. 

Los localizadores apicales integrados en los motores endodónticos generalmente brindan mediciones exactas de la longitud de trabajo. Estudios previos muestran que estos dispositivos pueden mantener el límite apical de manera efectiva durante la preparación del conducto radicular, con una precisión comparable a la de los localizadores de ápice independientes como Root ZX II y VDW Gold ^11^^,^^21^. A pesar de ello, un estudio reciente encuestó a 297 odontólogos e investigó sobre sus perspectivas y experiencias sobre el uso de localizadores apicales electrónicos, y su integración con motores endodónticos. Los resultados mostraron que, si bien el 95,6% de los participantes manifestó preferencia por la determinación electrónica de la longitud de trabajo, solo el 39,7% utilizaba motores con localizador integrado. Incluso entre los endodoncistas, el uso de la instrumentación integrada fue limitado, y muchos profesionales prefirieron confirmar la longitud de trabajo electrónica de forma pasiva antes y después de la preparación, en lugar de confiar plenamente en la función integrada durante la instrumentación ^22^. 

En el presente estudio, se planteó como objetivo evaluar la posible repercusión del uso de diferentes tipos de limas (manuales o mecanizadas) a la hora de utilizar los diferentes localizadores incorporados a motores. De manera global, pudo observarse que las limas manuales, especialmente las utilizadas con los localizadores Root ZX mini (Morita) y Endo motor SCM-01 (Fanta), mostraron mayor exactitud (menor error absoluto), así como menor variabilidad en la determinación de la longitud radicular. 

De acuerdo con los resultados, la determinación de la longitud radicular parece ser más exacta cuando se utiliza una lima manual, ya que el movimiento controlado minimiza el riesgo de sobreinstrumentación del foramen. En un estudio previo ^11^, donde se compararon las funciones de los dispositivos MM Control y Root ZX II, aunque no hubo diferencias significativas en las medias de las mediciones de longitud electrónica (EAL) o apical automatizada (AAR), la función EAL del MM Control presentó un mayor porcentaje de lecturas sobreextendidas (>1,01 mm más largas) en comparación con la longitud real (AL). Además, la función AAR mostró un límite apical aceptable en el 83,3% de los casos con Root ZX II y en el 77,8% con MM Control, destacando que las limas rotatorias pueden influir en la precisión de las mediciones y en la probabilidad de lecturas sobreextendidas. Otro reciente estudio *in vitro*^23^ evaluó la eficacia del motor Endus Duo Gnatus para determinar la longitud del conducto radicular mediante dos funciones: el modo de localizador electrónico (M2) con limas manuales de acero inoxidable, y el modo *autostop* (M3) durante la instrumentación rotatoria con limas de níquel-titanio (NiTi). Se compararon tres grupos: uno con lima manual de acero inoxidable (control), otro con instrumentación rotatoria activa (NiTi RI), y un tercero con colocación manual de lima NiTi (NiTi MUI). Los resultados mostraron que tanto el grupo control como el grupo NiTi RI lograron mediciones cercanas a la longitud real del conducto, mientras que el grupo NiTi MUI presentó mediciones significativamente más cortas (p < 0,001). Los autores concluyeron que el uso del motor en modo rotatorio (NiTi RI) ofrece resultados aceptables, pero la medición manual con limas NiTi puede comprometer la precisión.

Uno de los localizadores apicales más investigados es el Root ZX. En un estudio realizado por Aguiar *et al*., se analizó la exactitud de las tres versiones del dispositivo: Root ZX, Root ZX II y Root ZX mini. Los autores concluyeron que los tres modelos mostraron una precisión similar y clínicamente aceptable para la medición de la longitud del conducto radicular a nivel del foramen apical. Las principales diferencias entre los modelos radican en las funcionalidades adicionales, destacándose el Root ZX mini por ser una versión compacta con la capacidad de integrarse a un motor para la instrumentación mecánica, lo que ofrece ventajas prácticas en términos de versatilidad y portabilidad ^24^. Por otro lado, Khan *et al*. ^9^ comparaban la efectividad del Endo Radar Plus Motor y del Root ZX usando limas manuales donde, al igual que en el presente trabajo, el Root ZX mini fue el que proporcionó medidas más exactas para la determinación de la longitud del conducto ^9^^,^^25^. En otro estudio, Ramachandran *et al*. compararon el Root ZX y el Endo motor de Fanta, y concluyeron que ambos tenían el mismo nivel de eficiencia ^26^.

Una de las principales limitaciones del estudio fue la dificultad para unificar el tipo de pieza dentaria utilizada, ya que no siempre fue posible acceder a muestras homogéneas con características clínicas similares. Además, el tamaño muestral relativamente reducido podría limitar la generalización de los resultados. No obstante, al tratarse de una investigación in vitro, se logró mantener un mayor control sobre las variables experimentales, lo que permitió obtener datos comparables y consistentes. En este contexto, el estudio fue concebido como piloto, con el propósito de explorar la exactitud de diferentes combinaciones de localizadores apicales y tipos de limas. A pesar de las limitaciones mencionadas, los hallazgos aportan evidencia preliminar que puede servir como base para futuras investigaciones con un enfoque más amplio y un mayor número de muestras. 

La relevancia de este estudio se centra en la identificación de factores determinantes para lograr mediciones exactas de la longitud radicular, un elemento esencial en el éxito de los tratamientos endodónticos. Los resultados evidencian que las limas manuales ofrecen una exactitud superior en comparación con las mecanizadas, lo cual puede ayudar a prevenir errores como la sobreinstrumentación o subinstrumentación, que podrían impactar negativamente en el tratamiento. Asimismo, los dispositivos Root ZX mini y el Endo motor SCM-01 (función localizador apical) demostraron una mayor exactitud al utilizar limas manuales, posicionándose como opciones preferibles en situaciones donde la exactitud es fundamental. Por otro lado, la similitud en los resultados de los localizadores al emplear limas mecanizadas indica que cualquiera de estos equipos puede ser adecuado en procedimientos con instrumentos mecanizados. Estos hallazgos resaltan la importancia de considerar tanto el tipo de instrumento como la modalidad de activación del localizador en la práctica endodóntica.

## CONCLUSIÓN

Los resultados del presente estudio indican que la medición de la longitud radicular fue más exacta y con menor variabilidad cuando los localizadores apicales fueron utilizados con limas manuales, en comparación con las limas mecanizadas accionadas por motores endodónticos. En particular, los dispositivos Root ZX mini y Endo Motor SCM-011 mostraron un desempeño superior al emplearse con limas manuales.

## Financiación:

Financiado por los Fondos Concursables de la Universidad del Pacifico.
